# Hybrid liposomes inhibit tumor growth and lung metastasis of murine osteosarcoma cells

**DOI:** 10.1002/cam4.67

**Published:** 2013-03-22

**Authors:** Hideki Kitajima, Yuji Komizu, Hideaki Ichihara, Koichi Goto, Ryuichi Ueoka

**Affiliations:** Division of Applied Life Science, Graduate School of Engineering, Sojo UniversityKumamoto, Japan

**Keywords:** Apoptosis, hybrid liposomes, invasion, metastasis, osteosarcoma

## Abstract

Antitumor effects of hybrid liposomes (HL) composed of l-α-dimyristoylphosphatidylcholine (DMPC) and polyoxyethylene(23) dodecyl ether (C_12_(EO)_23_) on the metastatic growth of murine osteosarcoma (LM8) cells were investigated in vitro and in vivo. Remarkable inhibitory effects of HL-23 on the growth of LM8 cells were obtained through the induction of apoptotic cell death in vitro. It was also indicated that HL-23 should dramatically suppress the invasion of LM8 cells and the formation of filopodia on the cell surface in vitro. Furthermore, significantly high therapeutic effects were observed in the homograft mouse models of LM8 cells with lung metastasis after the treatment with HL-23 in vivo. That is, the histological analysis demonstrated that the primary tumor growth of LM8 cells implanted subcutaneously into the mice was inhibited along with the induction of apoptosis. In addition, it was found that HL-23 significantly decreased the lung metastasis of LM8 cells in the mouse models through the inhibition of primary tumor invasion. These results suggest that HL-23 could be a novel agent for the chemotherapy of osteosarcoma.

## Introduction

Osteosarcoma (OS) is a highly aggressive and the most common primary malignant tumor of the bone that usually affects the large bones of the arm or leg [1, 2]. OS has a propensity for early distant spread with hematogenous metastases involving the lung [3, 4]. In general, the treatment of OS includes chemotherapy with doxorubicin/adriamycin or methotrexate and large-scale surgery; however, current chemotherapeutic protocols do not significantly increase the postsurgical 5-year survival rate of 50–70% [3, 5]. Therefore, new drugs that effectively inhibit growth and pulmonary metastasis of OS cells are needed for clinical treatments.

Hybrid liposomes (HL), first developed by Ueoka et al. [6], can be prepared by simply ultrasonicating a mixture of vesicular and micellar molecules in buffer solutions. HL-23 composed of l-α-dimyristoylphosphatidylcholine (DMPC) and polyoxyethylene(23) dodecyl ether (C_12_(EO)_23_) without any drugs have remarkable inhibitory effects on the growth of various tumor cells along with apoptosis in vitro [7], in vivo [8, 9], and clinical applications [10]. In addition, we have revealed a good correlation between membrane fluidity of HL composed of DMPC and C_12_(EO)_*n*_ (*n* = 4, 8, 10, 21, 23, 25) and their growth inhibitions for colorectal cancer cells in vitro [11]. We have also demonstrated a good correlation between fluidity of plasma membranes of various cancer cells and anticancer effects of HL in vitro [12]. Significantly, the HL distinguished cancer cells and normal cells which have higher and lower membrane fluidities, respectively, and fused and accumulated preferentially into cancer cells for human hepatocarcinoma [13] and human adult T-cell leukemia cells [14]. Recently, we have reported the inhibitory effects of HL-23 on the growth and invasion/migration of human OS (U-2 OS) cells along with apoptosis in vitro [15]. However, the therapeutic effects of HL on the metastatic growth of aggressive OS cells in vivo have not yet been elucidated.

In this study, we investigated the inhibitory effects of HL-23 composed of DMPC and C_12_(EO)_23_ on the growth and invasion of murine OS (LM8) cells in vitro. Furthermore, the therapeutic effects of HL-23 on OS in vivo were examined using homograft mouse models of LM8 cells with lung metastasis.

## Materials and Methods

### Preparation of hybrid liposomes

HL-23 were prepared by sonication of a mixture containing 90 mol% DMPC (NOF, Tokyo, Japan) and 10 mol% C_12_(EO)_23_ (Sigma Chemical, St. Louis, MO) in 5% glucose solution using a bath-type sonicator (VS-N300, VELVO-CLEAR, Tokyo, Japan) at 45°C with 300 W as described previously [15]. The sample solutions were filtered using a membrane filter with 0.20 μm pore size. The liposomes composed of only DMPC (DMPC liposomes) were prepared in the same manner as described above.

### Dynamic light scattering measurement

The size of HL-23 was measured with an electrophoretic light scattering spectrophotometer (ELS-8000, Otsuka Electronics, Osaka, Japan) using a He−Ne laser (633 nm) at a 90° scattering angle. The hydrodynamic diameter (*d*_hy_) of HL-23 was calculated using the Stokes–Einstein formula (eq. 1), where *κ* is Boltzmann constant, *T* is the absolute temperature, *η* is the viscosity, and *D* is the diffusion coefficient:


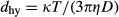
(1)

### Cell culture

Metastatic murine osteosarcoma cell line (LM8) was obtained in October 2011 from the cell bank Riken Bioresource Center (Saitama, Japan) [16] and frozen as original stocks in October 2011. LM8 cells were cultured in minimum essential medium (MEM) (Gibco, Gaithersburg, MD) supplemented with 10% fetal bovine serum (FBS), penicillin (100 units/mL), and streptomycin (100 units/mL). The cells were cultivated under standard culture conditions (95% humidified atmosphere of 5% CO_2_ at 37°C).

### Assessment of growth inhibition in vitro

The inhibitory effects of HL-23 on the growth of LM8 cells were examined on the basis of WST-8 (2-(2-methoxy-4-nitrophenyl)-3-(4-nitrophenyl)-5-(2,4-disulfophenyl)-2*H*-tetrazolium, monosodium salt) assay (Cell Counting Kit-8, Dojindo Laboratories, Kumamoto, Japan) [17] in vitro. LM8 cells (2.0 × 10^4^ cells/mL) were seeded in 96-well plates and cultured in a 5% CO_2_ humidified incubator at 37°C. After 24 h, HL-23 was added into each well and the plates were incubated for 24 h. Then, the WST-8 solution was added and the cells were incubated for 3 h. The absorbance at a wavelength of 450 nm was measured with a spectrophotometer (VERSAmax, Molecular Devices, Sunnyvale, CA). The inhibitory effects of HL-23 on the growth of LM8 cells were evaluated by *A*_mean_/*A*_control_, where *A*_mean_ and *A*_control_ denote the absorbance of water-soluble formazan in the presence and absence of HL-23, respectively.

### Hoechst 33342 staining assay

The fluorescent dye Hoechst 33342 (Dojindo Laboratories) was used to observe the apoptotic cells in vitro [18]. LM8 cells (2.0 × 10^4^ cells/mL) were seeded in 96-well plates, preincubated for 24 h, and treated with 5% glucose solution (control) or HL-23 (250 μmol/L for DMPC). After the incubation for 24 h, the cells were stained with Hoechst 33342 solution (10 μg/mL) for 10 min at room temperature. The stained cells were then observed using a fluorescence microscope (EVOSfl, Advanced Microscopy Group, Bothell, WA) with DAPI light cube (configured with an excitation filter of 357/44 nm and emission filter of 447/60 nm). The apoptotic LM8 cells, which were characterized by the nuclear chromatin condensation and/or nuclear fragmentation, were counted and the percentage of apoptotic cells was calculated.

### Invasion assay in vitro

Invasion of LM8 cells in vitro was examined using a BioCoat™ Matrigel™ invasion chamber (8 μm pore size) (BD Biosciences, Bedford, MA) according to the manufacturer's instructions. Briefly, 0.45 mL of LM8 cells (4.0 × 10^4^ cells/mL) were suspended in a serum-free medium containing either 5% glucose solution (control) or HL-23 (50, 100, 200 μmol/L for DMPC), and the cell suspension was added to the cell culture insert of a BioCoat™ Matrigel™ invasion chamber. Subsequently, 0.7 mL of medium supplemented with 10% FBS was added to the outer chamber as a chemoattractant. The cells were then incubated in a 5% CO_2_ humidified incubator at 37^ο^C for 24 h. To quantify the invasiveness of LM8 cells, the noninvading cells were removed from the upper surface of the PET membrane coated with Matrigel Matrix by scrubbing gently with a cotton-tipped swab. The cells on the lower surface of the membrane, which had invaded through the Matrigel layer and 8 μm membrane pores, were fixed with ethanol and stained with hematoxylin (Wako Pure Chemical industries, Osaka, Japan). The number of stained cells was counted by microscopic observation (Eclipse TE300, Nikon, Tokyo, Japan), and the average number per unit area (mm^2^) of the membrane was calculated.

### Total internal reflection fluorescence microscopy

To assess the filopodia formation of LM8 cells, the cells were analyzed by total internal reflection fluorescence (TIRF) microscopy. LM8 cells (5.0 × 10^4^ cells/mL) were seeded in glass bottom dishes and incubated for 24 h. Subsequently, the cells were treated with either 5% glucose solution (control) or HL-23 (50 μmol/L) for 3 h. Next, the cells were washed with PBS (−) and fixed with a 10% formaldehyde solution for 10 min at room temperature. After washing with PBS (−), the cells were permeabilized with 0.1% Triton X-100 for 10 min at room temperature. After that, the cells were incubated with 0.3 units/mL of rhodamine-labeled phalloidin (Molecular Probes, Eugene, OR) for 30 min. The stained cells were observed with a TIRF microscope system (IX71, Olympus, Tokyo, Japan) equipped with an air-cooled CCD camera (EM-CCD C9100-13, Hamamatsu Photonics, Hamamastu, Japan), and the average number of filopodia per cell was calculated.

### Assessment of therapeutic effects in vivo

The mice were handled in accordance with the guidelines for animal experimentation set out in Japanese law. The animal studies were approved by the Committee on Animal Research of Sojo University. BALB/c-R/J mice were kindly provided by Professor Okada (Kumamoto University, Japan) [19]. The mice were randomly grouped on the basis of body weight by the stratified randomization method. The number of mice was five in each group. LM8 cells (2.0 × 10^6^ cells) were implanted subcutaneously into the dorsal flank of the mice [20]. HL-23 (Dose: 203 mg/kg/day for DMPC, 41 mg/kg/day for C_12_(EO)_23_) was intravenously administered once each day for 14 days after the inoculation of LM8 cells. The primary tumors and lungs were resected from the anesthetized mice after 14 days of inoculation of LM8 cells and fixed in 10% formalin solution. The primary tumors and lungs were embedded in paraffin and sectioned at 5 μm of thickness. The sections of primary tumors and lungs were stained with hematoxylin and eosin (HE) and observed with an optical microscope (Nikon TS-100, Tokyo, Japan). The tumor dimensions were estimated using an image analysis software ImageJ (Version 1.46r, National Institutes of Health, Bethesda, MD).

### TUNEL method

Detection of apoptotic cells in vivo was performed on the basis of the TUNEL method using an in situ apoptosis detection kit (S7100, Chemicon International, Billerica, MA). The primary tumors of LM8 cells were resected from anesthetized homograft mouse models after the intravenous administration of HL-23 once each day for 14 days and fixed in 10% formalin solution. The paraffin-embedded sections were cut, dewaxed in xylene, and rehydrated through a series of ethanol to water. The sections were incubated with proteinase K for 15 min at room temperature and endogenous peroxidase was blocked with PBS (−) containing 2% H_2_O_2_ for 5 min. The sections were then incubated with a solution of digoxigenin-conjugated nucleotides and terminal deoxynucleotidyl transferase (TdT) at 37°C for 60 min. Subsequently, the antidigoxigenin antibody was applied and the sections were incubated for 30 min at room temperature. Detection of the antigen–antibody link was made through immunoperoxydase followed by 3,3′-diaminobenzidine (DAB) chromogen. The sections were counterstained with 5% methyl green, rinsed in distilled water, and observed with an optical microscope.

### Assessment of invasion in vivo

BALB/c-R/J mice were randomly grouped on the basis of body weight by the stratified randomization method. The number of mice was three in each group. LM8 cells (2.0 × 10^6^ cells) were implanted subcutaneously into the dorsal flank of mice [20]. HL-23 (Dose: 203 mg/kg/day for DMPC, 41 mg/kg/day for C_12_(EO)_23_) was intravenously administered once each day for 7 days after the inoculation of LM8 cells. The primary tumors in the subcutaneous tissues were resected from the anesthetized mice after 7 days of inoculation of LM8 cells and fixed in 10% formalin solution. The primary tumors were embedded in paraffin and sectioned at 5 μm of thickness. The sections were stained with HE and the invasion of primary tumors into the peritoneum was observed by an optical microscope.

### Statistical analysis

Results are presented as mean ± SE. Data were statistically analyzed using Student's *t*-test. A *P* value of ≤0.05 was considered to represent a statistically significant difference.

## Results

### Physical properties of HL-23

**Figure 1:**
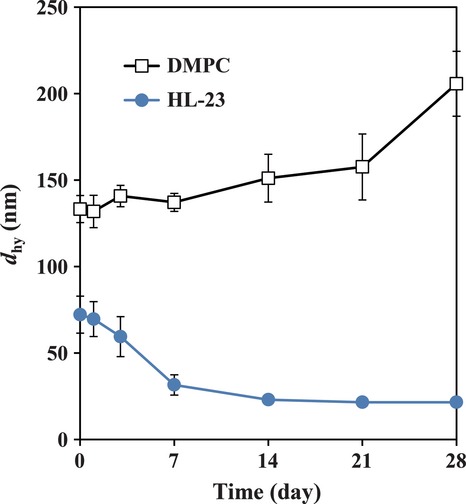
Time course of hydrodynamic diameter (*d*_hy_) change for HL-23 at 25°C. Error bars indicate SE for four individual experiments. DMPC liposomes: [DMPC] = 10 mmol/L, HL-23: [DMPC] = 10 mmol/L, [C_12_(EO)_23_] = 1.1 mmol/L. HL, hybrid liposomes; SE, standard error; DMPC, l-α-dimyristoylphosphatidylcholine; C_12_(EO)_23_, polyoxyethylene(23) dodecyl ether.

HL-23 were prepared by sonication of a mixture containing 90 mol% DMPC and 10 mol% C_12_(EO)_23_ in 5% glucose solution and the morphology of HL-23 was examined on the basis of dynamic light scattering measurements. As shown in Figure 1, the *d*_hy_ of HL-23 was about 40 nm, which was preserved for a period that remained stable for 4 weeks. On the other hand, *d*_hy_ of DMPC liposomes was larger than that of HL-23, and gradually increased with time and became over 200 nm after 4 weeks.

### Hybrid liposomes inhibit growth of OS cells in vitro

**Figure 2:**
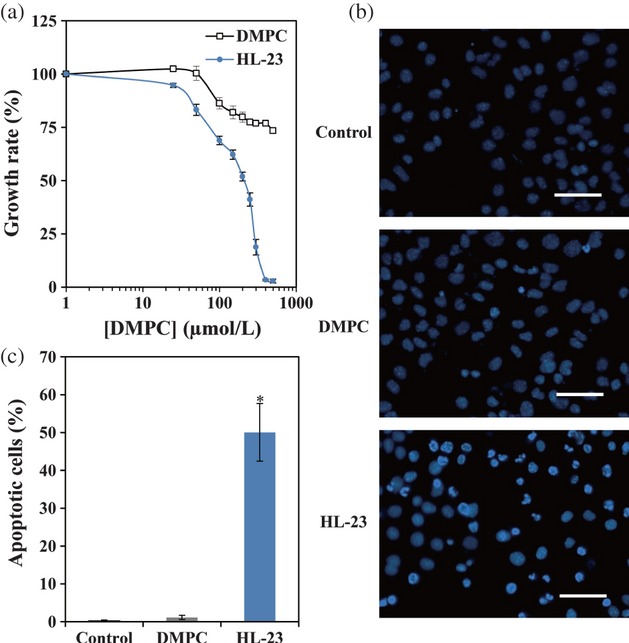
Inhibitory effects of HL-23 on the growth of LM8 cells in vitro. (a) Concentration dependence of HL-23 on the growth of LM8 cells in the culture medium for 24 h. Error bars indicate SE for four individual experiments. (b) Micrographs of LM8 cells stained with Hoechst 33342 after the treatment with HL-23 for 24 h by fluorescence microscopy. Scale bars: 50 μm. (c) Percentage of apoptotic LM8 cells. Error bars indicate SE for four individual experiments. *P < 0.05 to control. Control: 5% glucose solution, DMPC liposomes: [DMPC] = 250 μmol/L, HL-23: [DMPC] = 250 μmol/L, [C_12_(EO)_23_] = 28 μmol/L. HL, hybrid liposomes; LM8, murine osteosarcoma; SE, standard error; DMPC, l-α-dimyristoylphosphatidylcholine; C_12_(EO)_23_, polyoxyethylene(23) dodecyl ether.

First, we examined the inhibitory effects of HL-23 on the growth of LM8 cells on the basis of WST-8 assay in vitro. The results are shown in Figure 2a. HL-23 inhibited the growth of LM8 cells in a dose-dependent manner with a 50% inhibitory concentration (IC_50_) value of 205 μmol/L for DMPC, whereas DMPC liposomes slightly inhibited the growth of LM8 cells in the concentration range of 0–500 μmol/L for DMPC (IC_50_ >500 μmol/L). In our previous study, it was reported that HL-23 induced apoptotic cell death in various tumor cells including human OS (U-2 OS, MG-63) cells in vitro [11–15]. Here, we observed the induction of apoptosis by HL-23 in LM8 cells on the basis of Hoechst 33342 staining assay. The fluorescence micrographs of LM8 cells stained with Hoechst 33342 are shown in Figure 2b. The nuclear chromatin condensation and nuclear fragmentation were observed in LM8 cells treated with HL-23, indicating that HL-23 induced apoptosis toward LM8 cells. On the other hand, the apoptotic cells were barely detected in the control cells and DMPC liposomes-treated cells. The percentages of apoptotic cells were 0.22 ± 0.21% for the control cells, 1.1 ± 0.6% for DMPC liposomes-treated cells, and 50.1 ± 7.6% for HL-23-treated cells (Fig. 2c).

### Hybrid liposomes suppress invasion of OS cells in vitro

**Figure 3:**
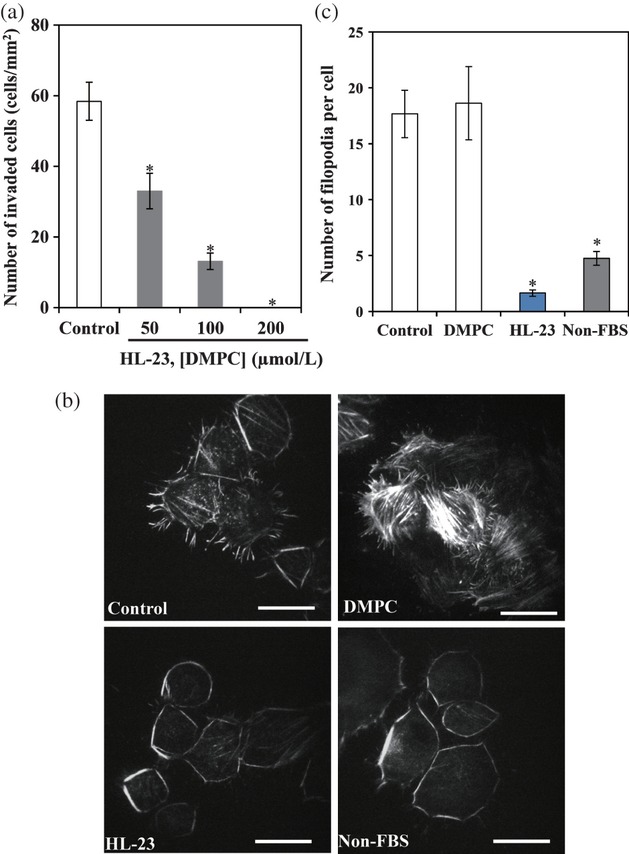
Inhibitory effects of HL-23 on the invasion of LM8 cells in vitro. (a) In vitro invasion assay. Invaded cell number of LM8 cells after the treatment of HL-23 for 24 h. Error bars indicate SE for three individual experiments. *P < 0.05 to control (5% glucose solution). (b) Micrographs of LM8 cells stained with rhodamine–phalloidin after the treatment of HL-23 for 3 h by TIRF microscopy. Scale bars: 20 μm. (c) Number of filopodia on the surface of LM8 cells. Error bars indicate SE for 8–14 individual experiments. *P < 0.05 to control. Control: 5% glucose solution, DMPC liposomes: [DMPC] = 50 μmol/L, HL-23: [DMPC] = 50 μmol/L, [C_12_(EO)_23_] = 5.6 μmol/L, non-FBS: LM8 cells were incubated in the serum-starved medium for 24 h. HL, hybrid liposomes; LM8, murine osteosarcoma; SE, standard error; TIRF, total internal reflection fluorescence; DMPC, l-α-dimyristoylphosphatidylcholine; C_12_(EO)_23_, polyoxyethylene(23) dodecyl ether; FBS, fetal bovine serum.

Second, we observed the effects of HL-23 on the invasion of LM8 cells using a BioCoat™ Matrigel™ invasion chamber in vitro. Figure 3a shows that the number of invaded LM8 cells was decreased by increasing the concentration of HL-23 (IC_50_ = 56 μmol/L). Interestingly, HL-23 significantly reduced the invasion of LM8 cells at the concentration of 50 μmol/L, though HL-23 hardly inhibited the growth of LM8 cells at the same concentration (Figs. 2a and 3a). On the other hand, it is well known that filopodia on the surface of tumor cells play an important role in the migration and invasion into the peripheral tissue [21, 22]. It has also been reported that the degree of filopodia formation of LM8 cells is closely related to the migrative/invasive and metastatic potentials [16, 23]. Therefore, the effects of HL-23 on the filopodia formation of LM8 cells were observed by TIRF microscopy in vitro. As shown in Figure 3b, LM8 cells had a few filopodia on the surface after the treatment of HL-23 (50 μmol/L) for 3 h, while the control and DMPC liposomes-treated LM8 cells had many filopodia. A similar tendency was observed in LM8 cells incubated in the serum-starved (non-FBS) medium for 24 h. The number of filopodia on the surface of LM8 cells was counted microscopically, and the results are shown in Figure 3c. The average numbers of filopodia per cell were 17.7 ± 2.1 for the control cells, 18.6 ± 3.3 for DMPC liposomes-treated cells, 1.6 ± 0.3 for HL-23-treated cells, and 4.8 ± 0.7 for non-FBS cells.

### Hybrid liposomes inhibit primary tumor growth of OS cells in vivo

**Figure 4:**
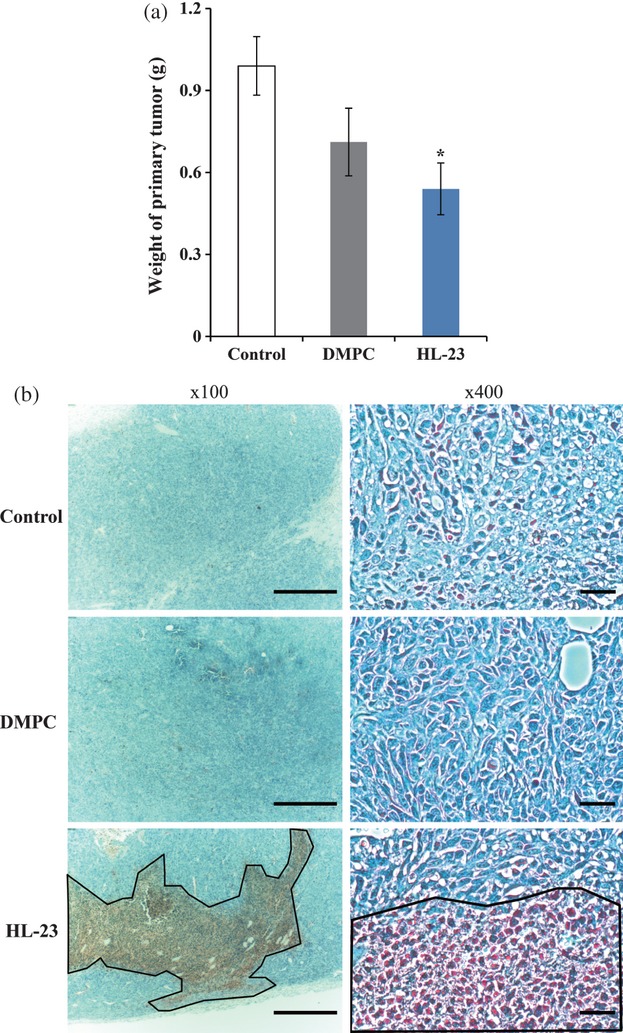
Inhibitory effects of HL-23 on the primary tumor growth of LM8 cells in homograft mouse models in vivo. (a) Weight of subcutaneous primary tumor of the mouse models treated with HL-23 for 14 days. Error bars indicate SE (*n* = 6 per group).*P < 0.05 to control. (b) Representative micrographs of subcutaneous primary tumor resected from the mouse models treated with HL-23 for 14 days by TUNEL method. Scale bars: 500 μm (×100), 50 μm (×400). Dotted enclosures indicate apoptotic tumor cells. Control: 5% glucose solution, DMPC liposomes: 203 mg/kg/day, HL-23: 203 mg/kg/day for DMPC, 41 mg/kg/day for C_12_(EO)_23_. HL, hybrid liposomes; LM8, murine osteosarcoma; SE, standard error; DMPC, l-α-dimyristoylphosphatidylcholine; C_12_(EO)_23_, polyoxyethylene(23) dodecyl ether.

With respect to the antitumor effects of HL-23 on the metastatic OS in vivo, we investigated the effects of HL-23 on the growth of the primary tumor in the homograft mouse models of LM8 cells with lung metastasis. HL-23 was administered into the caudal vein of BALB/c-R/J mice once a day for 14 days after the LM8 cells were subcutaneously inoculated into the dorsal flank of mice. Inhibitory effects of HL-23 on the primary tumor growth of LM8 cells in vivo are shown in Figure 4a. The average tumor weight (0.45 ± 0.10 g) of HL-23-treated mice in vivo was significantly decreased in comparison with that of the control mice (0.99 ± 0.11 g) (*P* = 0.011). On the other hand, DMPC liposomes-treated mice had a tendency to lower the tumor weight (0.71 ± 0.12 g) as compared with the control mice; however, these values did not reach statistical significance (*P* = 0.136). In addition, the induction of apoptosis into the primary tumor of LM8 cells was observed in the mouse models treated with HL-23 on the basis of histological analysis by the TUNEL method. The results are shown in Figure 4b. A significant number of apoptotic cells appeared brown in the tumor tissue of HL-23-treated mice. In contrast, the apoptotic cells were not observed in those of the control mice and DMPC liposomes-treated mice.

### Hybrid liposomes inhibit lung metastasis of OS cells in vivo

**Figure 5:**
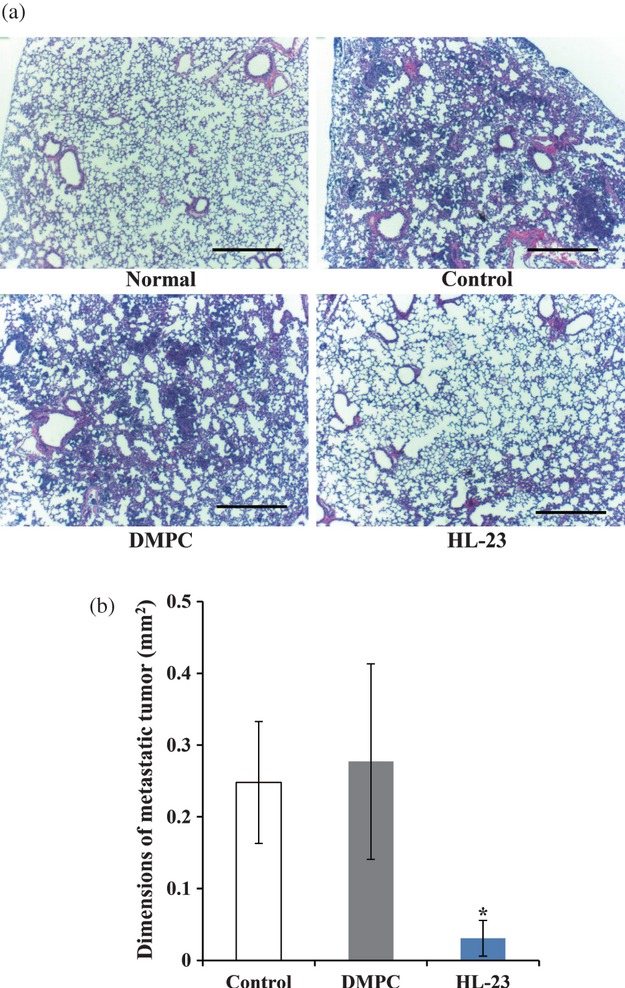
Inhibitory effects of HL-23 on the lung metastasis of LM8 cells in homograft mouse models in vivo. (a) Micrographs of lung tissue resected from the mouse models treated with HL-23 for 14 days by HE staining method. Scale bars: 500 μm. (b) Dimensions of metastatic tumor in the lung tissue of mouse models treated with HL-23 for 14 days. Error bars indicate SE (*n* = 5 per group). *P < 0.05 to control. Control: 5% glucose solution, DMPC liposomes: 203 mg/kg/day, HL-23: 203 mg/kg/day for DMPC, 41 mg/kg/day for C_12_(EO)_23_. HL, hybrid liposomes; LM8, murine osteosarcoma; HE, hematoxylin and eosin; SE, standard error; DMPC, l-α-dimyristoylphosphatidylcholine; C_12_(EO)_23_, polyoxyethylene(23) dodecyl ether.

**Figure 6:**
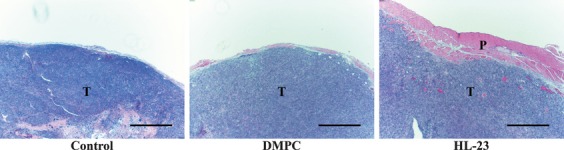
Micrographs of the subcutaneous tumor of LM8 cells resected from homograft mouse models treated with HL-23 for 7 days by HE staining method. The number of mice was three in each group. Scale bars: 500 μm. Control: 5% glucose solution, DMPC liposomes: 203 mg/kg/day, HL-23: 203 mg/kg/day for DMPC, 41 mg/kg/day for C_12_(EO)_23_. T, tumor; P, peritoneum; HL, hybrid liposomes; LM8, murine osteosarcoma; HE, hematoxylin and eosin; DMPC, l-α-dimyristoylphosphatidylcholine; C_12_(EO)_23_, polyoxyethylene(23) dodecyl ether.

In order to gain further insight into the antitumor effects of HL-23 on the metastatic growth of OS in vivo, we examined the effects of HL-23 on the lung metastasis of LM8 cells in the homograft mouse models in an autopsy. The lungs were removed from anesthetized homograft mouse models after the treatment with HL-23 for 14 days and observed with an optical microscope by HE staining method. The results are shown in Figure 5a. The enlarged tumor nodes of metastatic tumor were observed in the lung tissues of control mice and DMPC liposomes-treated mice. On the other hand, markedly fewer metastatic tumors were detected in the lung tissue of HL-23-treated mice. The dimensions of metastatic tumors in the lungs of mouse models were estimated using an image analysis software ImageJ. The results are shown in Figure 5b. The dimensions of metastatic tumors of the control mice and DMPC liposomes-treated mice were 0.25 ± 0.09 mm^2^ and 0.28 ± 0.14 mm^2^, respectively, whereas that of HL-23-treated mice was 0.031 ± 0.025 mm^2^ (*P* < 0.05). Furthermore, the inhibitory effects of HL-23 on the invasion of the primary tumor in the homograft mouse models of LM8 cells were observed on the basis of the histological analysis. HL-23 was intravenously administered once each day for 7 days after the inoculation of LM8 cells. The primary tumor of LM8 cells in the subcutaneous tissue was stained with HE and observed by an optical microscope. The results are shown in Figure 6. The peritoneum on the outside of subcutaneous tumors was drastically decreased in the control mice and DMPC liposomes-treated mice as compared with HL-23-treated mice, though the histolysis of peritoneum (peritoneal muscle) was observed in the boundary region between the tumor and the peritoneum for HL-23-treated mice.

## Discussion

OS accounts for about 60% of bone sarcomas in the pediatric age group [24]. Despite advances in surgery, radiotherapy, and chemotherapy, long-term survival rates of OS have stagnated at approximately 65%, primarily due to its metastasis with distant spread mostly to the lungs in about 80% of patients [25, 26]. Therefore, new drugs that effectively inhibit growth and pulmonary metastasis of OS cells are needed for clinical treatments. On the other hand, the LM8 cell line, originated from the murine osteosarcoma cell line [16], is one of the most commonly used cell lines to test antimetastatic compounds for OS [27–30]. LM8 cells metastasize to the lungs with 100% efficiency after the inoculation in mice [27–29]. Consequently, this homograft model is suitable for investigation of tumor growth and lung metastasis of OS in vivo. In our previous study, we have reported that the HL-23 composed of 90 mol% DMPC and 10 mol% C_12_(EO)_23_ inhibited the growth of human OS (U-2 OS, MG-63) cells along with apoptosis in vitro [15]. However, the therapeutic effects of HL-23 for lung metastasis models of OS cells in vivo have not yet been elucidated. In this study, we have demonstrated that HL-23 inhibit the growth and metastasis of the murine OS cell line LM8 in vitro and in vivo.

First, we examined the inhibitory effects of HL-23 on the growth of LM8 cells on the basis of WST-8 assay in vitro. The IC_50_ values of HL-23 and DMPC liposomes for the growth of LM8 cells were 205 μmol/L and more than 500 μmol/L, respectively (Fig. 2a). The IC_50_ value of HL-23 was less than half that of DMPC liposomes. In addition, the Hoechst 33342 staining assay indicated that HL-23 induced apoptosis in LM8 cells (Fig. 2b and c). These results indicate that HL-23 should be effective for inhibiting the growth of LM8 cells along with apoptosis as well as in the case of MG-63 and U-2 OS cells [15]. HL-23, being more fluid as compared with DMPC liposomes, showed strong inhibitory effects on the growth of U-2 OS and MG-63 cells [15] and human colon tumor (WiDr) cells [11]. The high inhibitory effects of HL-23 on the growth of LM8 cells should be closely related to the membrane fluidity of HL-23, probably due to the membrane fusion of HL-23 with the tumor cells [11–14]. Plausibly, HL-23 could fuse and accumulate into LM8 cell membranes, and inhibit the growth of LM8 cells through the induction of apoptosis.

Second, we observed the effects of HL-23 on the invasion of LM8 cells in vitro. Our results for the matrigel invasion assay demonstrated that HL-23 significantly reduced the invasion of LM8 cells at the concentration of 50 μmol/L, though the growth of LM8 cells was hardly inhibited at the same concentration of HL-23 (Figs. 2a and 3a). Additionally, TIRF microscopic observations showed that the filopodia formation was suppressed on the surface of LM8 cells after the cultivation in the medium containing HL-23 (50 μmol/L) for 3 h, as similarly observed in the medium without chemoattractants (non-FBS) (Fig. 3b and c). On the other hand, DMPC liposomes did not suppress the filopodia formation under the same conditions. In our previous study, it was indicated that the anti-invasive effects of HL-23 for U-2 OS and MG-63 cells were associated with the suppression of the migration/invasion and cell motility in vitro [15]. It is presumed that HL-23 could specifically fuse into the plasma membrane of LM8 cells, modulate the filopodia formation and cell motility in the cell membrane, and decrease the invasive potential of LM8 cells.

Furthermore, the in vivo inhibitory effects of HL-23 on the primary tumor growth of LM8 cells in the homograft mouse models with lung metastasis were investigated. As shown in Figure 4a, remarkable high inhibition effects of the primary tumor growth were obtained in the mouse models after the treatment of HL-23. In addition, the induction of apoptosis by HL-23 was observed in the primary tumor of the mouse models on the basis of histological analysis (Fig. 4b). These results indicate that HL-23 has remarkable inhibitory effects on the tumor growth of LM8 cells along with apoptosis in vivo as well as in the in vitro experiments of U-2 OS and MG-63 cells [15]. On the other hand, apoptotic cell death was not observed in the primary tumor of mouse models treated with DMPC liposomes, though the mice had a tendency to lower the tumor weight as compared with the control mice. In our previous studies, it was demonstrated that HL-23 can inhibit the growth of cancer cells through the cell cycle arrest at the G_0_/G_1_ phase along with apoptosis, whereas DMPC liposomes induced G_0_/G_1_ arrest of cancer cells under the same conditions in vitro [31]. The tumor weight loss in the mouse models treated with DMPC liposomes could be caused by the induction of cell cycle arrest. Furthermore, HL-23 used in this study have a *d*_hy_ ≤100 nm (Fig. 1), which should be suitable for avoiding the reticular endothelial system in vivo [32]. These results suggest that HL-23 given intravenously could accumulate and induce the apoptosis into the primary tumor of the mouse models in vivo. As for the pulmonary metastasis of OS, the effects of HL-23 on the lung metastasis of LM8 cells in the homograft mouse models were observed in an autopsy. As shown in Figure 5a, the lung of HL-23-treated mice was almost the same as that of the normal mice, though the enlarged tumor nodes of the metastatic tumor were observed in those of the control mice and DMPC liposomes-treated mice. Markedly fewer dimensions of metastatic tumors were confirmed in the lungs of the HL-23-treated mice (Fig. 5b). This result indicates that HL-23 should be effective for inhibiting the lung metastasis of LM8 cells in vitro. How do HL-23 inhibit the lung metastasis of LM8 cells in the mouse models? We attempted to examine the effects of HL-23 on the invasion of primary tumors in the mouse models of LM8 cells on the basis of the histological analysis. In the cases of DMPC liposomes-treated mice and the control mice, the peritoneum on the outside of subcutaneous tumors was decreased beyond recognition (Fig. 6). The observations suggest that the primary tumor degraded the peritoneum and infiltrated into the surrounding tissue leading to the pulmonary metastasis. Importantly, in the case of the HL-23-treated mice, the peritoneum on the outside of the subcutaneous tumor was maintained in the original form. As mentioned above, HL-23 can suppress the migration/invasion and motility of OS cells in vitro. It is plausible that HL-23 fused into LM8 cells, decreased the invasive potentials of LM8 cells, and inhibited the lung metastasis of LM8 cells in the mouse models in vivo. With regard to the safety of HL-23, no weight loss was observed in the mouse models of LM8 during the intravenous administration period of HL-23 for 14 day, as shown in Figure S1. Furthermore, no abnormal findings of HL-23 in weight changes, hematological and biochemical examinations have been observed in safety test using normal rats [10], mice [33], and mice with a carcinoma [8]. These results suggest that HL-23 would be a novel agent without severe side effects for the chemotherapy of OS.

In conclusion, we clearly demonstrated for the first time the remarkable inhibitory effects of drug-free HL-23 on the tumor growth and lung metastasis of OS cells in the mouse models in vivo. The noteworthy aspects are as follows: (a) HL-23 having a *d*_hy_ ≤100 nm were successfully preserved for a period of 4 weeks. (b) Remarkable inhibitory effects of HL-23 on the growth and invasion of LM8 cells were obtained in vitro. (c) HL-23 significantly inhibited the primary tumor growth of LM8 cells in the homograft mouse models along with apoptosis in vivo. (d) HL-23 drastically inhibited the lung metastasis of LM8 cells in the mouse models through the inhibition of invasion in vivo. It is attractive that HL-23 showed the significantly therapeutic effects on the metastatic growth of aggressive OS cells through not only the induction of apoptosis but also the inhibition of invasion. The results in this study could contribute to the development of chemotherapy for patients with OS in future clinical applications.

## Supporting Information

**Figure S1.** Body weight change of mouse models treated with HL-23 after the inoculation of LM8 cells. Control: 5% glucose solution, DMPC liposomes: 203 mg/kg/day, HL-23: 203 mg/kg/day for DMPC, 41 mg/kg/day for C_12_(EO)_23_. Data presented are mean ± SD (*n* = 7 per group). There was no significant difference between the control group and the group treated with HL-23.
