# Supplementary figures and images for: Hybrid liposomes inhibit tumor growth and lung metastasis of murine osteosarcoma cells
Source: Cancer Med. 2013 Mar 22;2(3):267–76. doi: 10.1002/cam4.67 (PMC3699838; doi:10.1002/cam4.67)

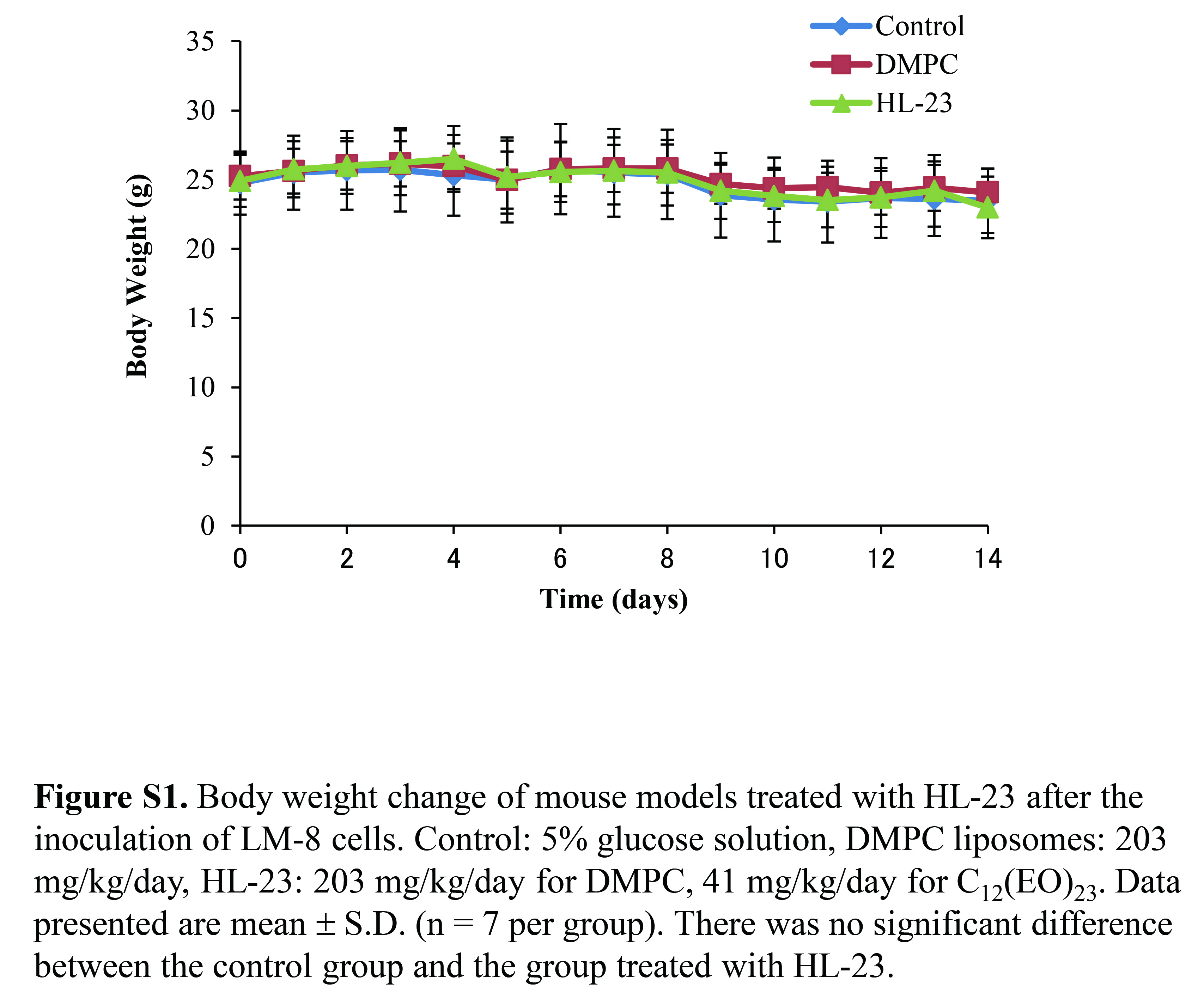

Supplement: Supplementary file 1 [file cam40002-0267-SD1.tif]
